# Suicidal Ideation in Patients With Multiple Sclerosis and Its Association With Clinical and Demographic Characteristics

**DOI:** 10.1002/brb3.70458

**Published:** 2025-04-01

**Authors:** Elham Bazmi, Amir Hossein Behnoush, Sharareh Eskandarieh, Abdorreza Naser Moghadasi, Samira Navardi, Saeideh Ayoubi, Behnam Behnoush, Mehdi Forouzesh, Mohammad Ali Sahraian

**Affiliations:** ^1^ Multiple Sclerosis Research Center, Neuroscience Institute, Tehran University of Medical Sciences, Neurology Department Sina Hospital Tehran Iran; ^2^ School of Medicine Tehran University of Medical Sciences Tehran Iran; ^3^ Department of Forensic Medicine Tehran University of Medical Sciences Tehran Iran; ^4^ Legal Medicine Research Center Legal Medicine Organization Tehran Iran

**Keywords:** anxiety, depression, multiple sclerosis, suicidal ideation

## Abstract

**Background:**

Chronic neurological diseases, such as multiple sclerosis (MS) are important risk factors for some psychological disorders, such as depression and consequently suicidal behavior. As suicidal ideation (SI) is one of the important factors in predicting suicide attempts and suicide deaths, the recognition of factors related to the occurrence of SI in patients with MS has high value.

**Objective:**

This study aims to assess the association between sociodemographic, psychological, and disease characteristics with SI in patients with MS.

**Methods:**

A sample of 400 subjects with MS was selected from a referral outpatient MS clinic in Tehran, Iran (2024). Participants completed a personal information questionnaire, the patient health questionnaire‐9 (PHQ‐9), the hospital anxiety and depression scale (HADS), and the beck scale for suicidal ideation (BSSI). Logistic regression models (univariate and multivariate) were conducted to determine the association between each variable and SI using odds ratio (OR) and 95% confidence interval (CI). Finally, the correlation between depression, anxiety, and the SI scale was measured by bivariate Pearson correlation analyses.

**Results:**

The sample consisted of 77% women, and the mean age was 36.91 years. Among them, 149 (37.2%) of cases were identified with suicidal ideation at some period in life. Findings in the multivariate regression model represented that an increase in age had a significant protective effect on SI (adjusted OR [AOR] 0.95, 95% CI 0.92 to 0.98, *p* = 0.003). Moreover, the history of previous suicide attempts doubled the odds of SI (AOR 2.18, 95% CI 1.05 to 4.54, P = 0.038). Finally, both depression score (measured by PHQ‐9) and anxiety score (measured by HADS) had significant associations with SI.

**Conclusion:**

People with MS are highly prone to suicidal thoughts and even suicide attempts; this should not be ignored in routine clinical visits due to other physical complaints. They need to be discussed with patients, and effective psychiatric interventions should be applied to reduce the risks.

## Graphical abstract

Summary of methodology, main findings, and conclusion for assessment of related factors to suicidal ideation in patients with multiple sclerosis (MS)

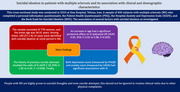

## 1 Introduction

Multiple Sclerosis (MS) is a chronic neurological disease that affects the central nervous system (CNS) and inflammatory processes. The breakdown of the myelin and, consequently, damage to the axons causes difficult‐to‐control and often disabling physical, cognitive, and emotional symptoms. Thus, people with MS face significant problems in coping with this chronic and unpredictable disease (Compston and Coles 2008; Inojosa et al. 2021).

The majority of patients with MS suffer from a series of symptoms, like sensory and motor loss, fatigue, impaired vision, difficulties with balance, pain, and cognitive impairment, in addition to depression, anxiety, and delirium, that affect many daily activities and decrease their physical, mental, and social quality of life and increased the risk of some comorbidities (Barin et al. 2018; Cehelyk et al. 2019; Weiss et al. 2005). Previous studies showed that early recognition and treatment of these disturbances could contribute to the reduction of disability and even to decreased mortality. One of the most important emotional and mood disorders in these patients is the increase in suicidal attempts (Raimo et al. 2021).

As suicidal ideation (SI) is one of the important factors in predicting suicide attempts and completed suicide, it is considered an index of other mental health problems; hence, the recognition of factors related to the occurrence of SI is important (Jobes and Joiner 2019). Some researchers showed that suicide in people with MS is approximately twice as high, and the prevalence of SI in people with MS is higher than in the general population (Kalb et al. 2019). So, the recognition of variables related to the occurrence of these thoughts is of high importance. It has been suggested that male sex, younger age, lower income, social isolation, the first five years of MS diagnosis, progressive disease subtype, earlier disease course, and higher levels of physical disability heighten the possibility of suicide (Feinstein and Pavisian 2017; Franklin et al. 2017; Viner et al. 2014). As MS progresses, higher levels of disability emerge, leading to an increase in the severity of depression and a decrease in the quality of life (QOL) (Barry et al. 2018; Tauil et al. 2018). On the other hand, healthcare providers and physicians mainly focus on physical disability in these patients, while mental health, psychological distress, and SI are usually ignored.

In Iran, a study was conducted on 234 MS patients during the COVID‐19 pandemic, which showed that SI had a prevalence of 46.5%, much of which could be attributed to the pandemic (Pouradeli et al. 2024). Another previous study also revealed that 30% of the patients with MS had suicidal thoughts (Sariaslani et al. 2021). However, these studies had low sample sizes or were conducted in special conditions, such as during the pandemic. Moreover, they did not assess the effect of anxiety on SI. As the bio psychosocial model suggests, the interaction between biological factors, psychological factors, and social factors shapes mental health outcomes, which could be the case for MS as well. To our knowledge, there are few updates and extensive research on the impact of socio‐demographic, physical, and mental characteristics in addition to the levels of psychiatric disturbances (depression and anxiety) on suicidal thoughts in patients with MS in Iran. Therefore, the present study is designed to investigate this association with the aim of reducing their disabilities through the administration of preventive policies, receiving psychotherapy, drug treatment, and other mental health‐related services, and preventing or lowering the risk of suicide in these patients.

## 2 Methods

### 2.1 Design and Study Participants

This cross‐sectional research was conducted on patients with MS referred to the outpatient MS Clinic of Sina Hospital (a referral hospital for MS patients) affiliated with Tehran University of Medical Sciences in 2024. The study was approved by the Ethics Committee of Tehran University of Medical Sciences, Tehran, Iran (IR.TUMS.NI.REC.1402.068), and was conducted in accordance with the Declaration of Helsinki. All participants had written informed consent.

Of 456 patients diagnosed with MS who were approached to participate in the study, 400 people agreed (response rate: 87.7%) and signed the informed consent. All included patients were at the age of 18–65 years and could read and write questionnaires and were allowed to interview with them. They met the McDonald MS diagnostic criteria by a neurologist. Exclusion criteria were chronic disease and pregnancy. Data collection was achieved by filling out the self‐reported questionnaire, semi‐structured interview, and neurological examination of the patients. The questionnaire contained the items concerning their clinical history, socio‐demographic characteristics, and four main measures, mentioned below.

### 2.2 Measures

The questionnaire contained items concerning their socio‐demographic characteristics, clinical history, and mental assessment:

#### 2.2.1 Personal Information Questionnaire

It consists of sociodemographic data (age, sex, marital status, education, job, current alcohol consumption, smoking (cigarette or waterpipe), history of suicide attempt, and use of psychiatric medications), MS disease characteristics on the basis of physician's diagnosis (MS type: Relapsing‐remitting MS and progressive MS), duration of illness, ambulatory disability, drugs used for MS treatment [no drugs, Ocrelizumab, Rituximab, Dimethyl fumarate, Fingolimod, and Interferon Beta] (Ghadiri et al. 2023).

#### 2.2.2 The Patient Health Questionnaire‐9 (PHQ‐9)

It was used as a self‐administered version of the PRIME‐MD diagnostic instrument to indicate depression severity and the presence of suicidal thoughts (Altura et al. 2016). While having its own limitations, this questionnaire is widely used as a measure of depression assessment and SI measurement in patients with chronic illnesses and MS (Beswick et al. 2022; Hind et al. 2016). In the PHQ‐9 questionnaire, which corresponds with DSM criteria for depression, items 1 to 8 evaluate depression and item nine assesses suicidal ideation in the patients. Each item score ranged from “zero” (not at all) to “three” (nearly every day). Scores less than five almost always signified minimal depressive disorder, scores of five to nine predominantly represented patients with mild depression, scores of 10 to 14 represented moderate depression, and scores of 15 to 19 showed moderately severe and 20 to 27 indicated severe depression (Kroenke et al. 2001). The Persian version of PHQ‐9 has been shown to have good reliability (Cronbach's alpha: 0.88) and validity in the Iranian population (Dadfar et al. 2018). The validity of item nine of the PHQ‐9 questionnaire has been confirmed as a screening tool to evaluate the risk of suicide mortality in addition to symptoms of depression in patients with neurological disorders (Louzon et al. 2016).

#### 2.2.3 The Hospital Anxiety and Depression Scale (HADS)

Physical symptoms have been removed from this questionnaire so it can be used to diagnose depression and anxiety in people with significant physical disease. In our study, the anxiety subscale of the HADS questionnaire was used for the assessment of anxiety. This was used to investigate the independent contribution of anxiety to SI in patients with MS and has been widely used for the measurement of anxiety in this population (Ellwardt et al. 2022; Giordano et al. 2011; Honarmand and Feinstein 2009). The HADS is a self‐report rating scale of 14 items on a four‐point Likert Scale (range 0–3). The score ranges from 0 to 21, with a higher score representing severe depressive symptomatology. The score 0–7 represents “normal,” 8–10 “mild,” 11–14 “moderate,” and 15–21 “severe.” In the present study, the cut‐off score of ≥8 and of ≥11 was used for HADS subscales (Pais‐Ribeiro et al. 2018; Stern 2014). Cronbach's alpha in our sample for the depression and anxiety subscale was 0.78 (Montazeri et al. 2003).

#### 2.2.4 Beck Scale for Suicidal Ideation (BSSI)

This instrument is a 19‐item questionnaire that evaluates the presence and intensity of SI in the last week before evaluation. Each item is scored from 0 to 2 and the total score is 0 to 38. In the screening part of assessing the SI score, if the patient's answer to the fifth item was positive (scores 1 and 2), the participant could answer the rest of the items; otherwise, the questionnaire is considered complete (Beck et al. 1988). The persian version of BSSI exhibited substantial internal consistency, as indicated by Cronbach's alpha value, which was α > 0.85 (Esfahani et al. 2015).

### 2.3 Statistical Analyses

Descriptive statistical analyses were carried out to show the mean and standard deviation *(SD)* for continuous variables and frequency and percent for categorical variables. For continuous variables, the mean difference was calculated as an effect size. In the analytical analysis, the differences in characteristics between the two groups (with or without SI) were examined using the chi‐square test for categorical variables and the t‐test for quantitative variables. A binary logistic regression model (univariate and multivariate) was conducted to determine the association between predictor variables and SI while adjusting for potential confounders. Crude and adjusted ORs with 95% confidence interval s (CIs) were reported. The assessment of multicollinearity was performed using the variance inflation factor (VIF), and model fit was evaluated to validate the regression models. In addition, bivariate Pearson correlation analyses were performed to explore the relationships between continuous variables (e.g., depression, anxiety, and disability scores) and the SI scale. Logistic regression was used to identify independent predictors of SI, while Pearson correlations provided insight into bivariate associations between continuous measures. All analyses were performed using SPSS software (version 27, IBM Corporation), and statistical significance was set at *p* < 0.05.″

## 3 Results

Among the total of 400 patients with a diagnosis of MS, 149 (37.2%) cases were identified to have SI using both the Beck questionnaire and item 9 of the PHQ‐9. The sample consisted of 77% women, and the mean age was 36.91 ± 8.22 years (range: 18–65 years). The highest rate of SI was between the ages of 30 and 35 years, with a mean difference between suicidal and non‐suicidal patients of 1.91 ± 0.85 years (*p* <0.05). Moreover, a one unit increase in age resulted in a decrease of 3% in the odds of SI (OR 0.97, 95% CI 0.95 to 0.99, *p* = 0.025). The married population also had a lower rate of SI (OR 0.59, 95% CI 0.37 to 0.92). The majority of patients [372 (93%)] were living with their families, while 86.5% were employed. Among the participants, 13.5% reported previous suicide attempts, which were 7.6% in the non‐SI group and 23.5% in the SI group. Patients with previous suicide attempts had significantly higher odds of SI (OR 3.75, 95% CI 2.07 to 6.96, *p* < 0.001). Similarly, those with a psychiatric drug consumption history had significantly higher odds of SI (OR 2.26, 95% CI 1.42 to 3.60, *p* = 0.035).

According to PHQ‐9, the mean ± SD depression score in the total of 400 patients was 10.79 ± 6.29 (moderate level) during the previous two weeks, and by considering HADS scores, the anxiety score was 9.8 ± 3.69 (mild level). The mean PHQ score in patients with MS who had SI was 14.83 ± 5.30, and in the non‐SI group was 8.40 ± 5.57 (mean difference 6.42 ± 0.56, *p* < 0.05). A one unit increase in the PHQ‐9 scale for depression resulted in an OR of 1.22 (95% CI 1.17 to 1.27, *p* < 0.001) in the crude model. Regarding the HADS anxiety score, patients with SI had a mean HADS of 11.62 ± 3.63, while the non‐SI group had an average of 8.72 ± 3.28 (mean difference 2.90 ± 0.35, *p*< 0.05). One unit increase in HADS score also increased the odds of SI by 26% (OR 1.26, 95% CI 1.19 to 1.35, *p* < 0.001). Demographic characteristics in patients based on suicidal ideation are shown in Table 1.

**TABLE 1:** Sociodemographic and psychiatric characteristics in patients based on suicidal ideation.

Variable	No suicidal ideation (*n* = 251)	Suicidal ideation (*n* = 149)	Total	OR (95% CI)	*P*‐value
Sex					
Male	60 (23.9)	32 (21.5)	92 (23)	1	0.577
Female	191 (76.1.)	117 (78.5)	308 (77)	1.15 (0.71‐1.88)	
Age	37.63 ± 8.55	35.71 ± 7.51	36.91 ± 8.22	0.97 (0.95‐0.99)	0.025^*^
Education level					
Preliminary	10 (4)	10 (6.7)	20 (5)	1	
High school diploma	54 (21.5)	24 (16.1)	78 (19.5)	0.44 (0.16‐1.2)	0.112
University degree	187 (74.5)	115 (77.2)	302 (75.5)	0.61 (0.25‐1.54)	0.293
Marital status					
Single	66 (26.3)	53 (35.6)	119 (19.55)	1	
Married	167 (66.5)	79 (53)	246 (61.5)	0.59 (0.37‐0.92)	0.021*
Other	18 (7.2)	17 (11.4)	35 (8.8)	1.18 (0.55‐2.51)	0.674
Living status					
Alone	13 (5.2)	16 (10.7)	28 (7)	1	
Living with family	238 (94.8)	133 (89.3)	372 (93)	0.45 (0.21‐0.97)	0.042*
Job					
Unemployed	25(10)	29(19.5)	54(13.5)	1	0.008*
Employed	226(90)	120(80.5)	346(86.5)	0.46 (0.26‐0.82)	
Smoking					
No	190 (75.7)	110 (73.8)	300 (75)	1	
Yes	61 (24.3)	39 (26.2)	100 (25)	1.10 (0.69‐1.75)	0.676
Alcohol consumption					
No	204 (81.3)	112 (75.2)	316 (79)	1	
Yes	47 (18.7)	37 (24.8)	84 (21)	1.43(0.88‐2.33)	0.148
History of suicide attempt					
No	232 (92.4)	114(76.5)	346(86.5)	1	
Yes	19 (7.6)	35(23.5)	54(13.5)	3.75 (2.07‐6.96)	**<**0.001*
Psychiatric drug consumption					
No	204 (81.3)	98 (65.8)	302(75.5)	1	
Yes	47 (18.7)	51 (34.2)	98(24.5)	2.26(1.42‐3.60)	0.035*
Psychiatric state					
PHQ‐9 Score (Depression)	8.40 ±5.57	14.83±5.30	10.79±6.29	1.22 (1.17‐1.27)	P < 0.001
HADS Score (Anxiety)	8.72±3.28	11.62±3.63	9.8±3.69	1.26 (1.19‐1.35)	P < 0.001

*Note*: Data are reported as mean ± standard deviation (*SD*) and number and percentage.

OR: Odds ratio; CI: Confidence interval.

*Statistically significant.

Table 2 shows disease characteristics and depression/anxiety in patients based on their SI status. Relapsing‐remitting MS was the most prevalent in both SI and non‐SI groups, accounting for 95 (63.75%) and 167 (66.6%), respectively. As indicated, there was no significant relationship between SI and the subtype of MS. Moreover, there was no difference between SI and non‐SI groups in terms of disease duration, ambulatory impairment, and the types of drugs used for MS treatment (*p* > 0.05). Finally, ambulatory impairment was associated with a higher rate of SI as shown by the chi‐square p‐value and the OR (2.10, 95% CI 1.22 to 3.62, *p* = 0.008).

**TABLE 2:** Disease characteristics in patients based on suicidal ideation.

Variables	No suicidal ideation (*n* = 251)	Suicidal ideation (*n* = 149)	Total	P‐value
Disease duration (year)	5.43±2.34	5.91±3.35	5.67±3.11	0.48
MS type				0.11
Relapsing‐remitting	167 (66.60)	95 (63.75)	262(65.5)	
Progressive	84 (33.40)	54 (36.22)	138(34.5)	
Ambulatory disability				0.007*
Yes	30 (11.9)	33 (22.1)	43 (10.8)	
No	221 (88.1)	116 (77.8)	264 (66)	
Drugs used for MS treatment				0.1
Without drugs	10 (4)	5 (3.8)	15 (3.8)	
Ocrelizumab	87 (34.7)	54(36.2)	141 (35.3)	
Riyuximab	50 (19.9)	37 (24.8)	87 (21.8)	
Dimethyl fumarate	24 (9.6)	13 (8.7)	37 (9.3)	
Fingolimodi	11 (4.4)	7 (4.7)	24 (6)	
Interferon Beta	20 (8)	4(2.7)	24(6)	
Others	49 (19.5)	29 (22)	78 (19.5)	

*Note*: Data are reported as mean ± standard deviation (*SD*) and number and percentage.

MS: Multiple sclerosis.

*Statistical significant.

As shown in Table 3, among patients with MS and SI, 51 (34.2%) had moderately severe depression (based on the PHQ‐9 questionnaire). This was followed by moderate depression in 34 patients (22.8%) and severe depression in 32 patients (21.5%). Regarding anxiety, 53 (35.6%) of those with SI had moderate anxiety, while 39 (26.2%) reported severe anxiety as measured by the HADS questionnaire. On the other hand, in the group without SI, the highest percentage of depression and anxiety levels were mild and normal, respectively. Both anxiety and depression levels had significant associations with SI among patients with MS.

**TABLE 3:** The Association between suicidal ideation with depression and anxiety levels in patients with MS.

Variables *n* (%)	No suicidal ideation (*n* = 251) *n* (%)	suicidal ideation (n = 149) *n* (%)	Pearson chi‐ square (p‐value)
Depression level			91.72 (<0.0001)
Minimal	72(28.7)	4 (2.7)	
Mild	80 (31.9)	28 (18.8)	
Moderate	61(24.3)	34 (22.8)	
Moderately severe	28 (11.2)	51 (34.2)	
Severe	10 (4)	32(21.5)	
Anxiety level			56.15 (<0.0001)
Normal	101 (40.2)	19 (12.8)	
Mild	78 (31.1)	38 (25.5)	
Moderate	55 (21.9)	53 (35.6)	
Severe	17 (6.8)	39 (26.2)	

According to the obtained results in the Pearson correlation analysis, there were significant correlations between depression and SI scores in the screening part of the BSSI (r (df) = 0.520, *p* < 0.001) and the whole scale of SI (r (df) = 0.466, *p* < 0.001). Also, the correlation between anxiety scores in the screening part of BSSI (r (df) = 0.442, *p* < 0.001) and the whole scale of SI (r (df) = 0.428, *p* < 0.001) was significant. So, by increasing psychiatric disturbances like depression and anxiety, the risk of suicidal thoughts increases.

In the final multivariate model, nonsignificant variables in the crude model were omitted (sex, education level, smoking, alcohol consumption), and adjusted OR for significant variables was evaluated, the results of which are summarized in Table 4. Based on our findings, while there was no significant association between SI and marital status, living status, employment, and psychiatric drug consumption, one unit increase in age resulted in a significant decrease in SI (adjusted OR [OR] 0.96, 95% CI 0.92 to 0.99, *p* = 0.003). Moreover, the history of suicide attempts had a positive association with SI (OR 2.16, 95% CI 1.05 to 4.54, *p* = 0.038). Finally, both the PHQ‐9 scale for depression and the HADS scale for anxiety had positive significant associations with SI.

**TABLE 4:** Adjusted OR for significant variables and in patients with MS.

Variables	Adjusted OR (CI 95%)	*P*‐value
Age	0.95 (0.92 ‐ 0.98)	0.003^*^
Marital status (ref: single)		
Married	0.69 (0.37‐1.27)	0.233
Others	0.82 (030‐2.15)	0.684
Living status (Ref: alone)	0.42 (0.16‐1.08)	0.074
Job (ref: unemployed)	0.98 (0.46–2.10)	0.96
Ambulatory impairment (ref: no)	1.76 (0.87 to 3.57)	0.117
History of suicide attempt (ref: no)	2.18 (1.05‐4.54)	0.038^*^
Psychiatric drug consumption (ref: no)	1.20 (0.7‐2.07)	0.501
Depression	1.18 (1.12–1.24)	**<**0.001*
Anxiety	1.08 (1.00 ‐1.18)	0.045*

*Note*: *Statistically significant.

Adjusted for significant variables in the univariate model.

## 4 Discussion

The aim of the present study was to evaluate the association between sociodemographic, psychological, and disease characteristics with SI in MS patients. To the base of our knowledge, this is the first study that has evaluated the association of SI levels with demographics, disease characteristics, and mode status levels in patients with MS in Iran. So, the findings attribute a novel addition to the literature. Based on our findings, patients with SI had a lower age, a previous history of suicidal attempts, and higher anxiety and depression scores. These highlight the need for the identification of those at higher risk of SI among the population with MS.

Our findings suggest that 37.6% of patients with MS had suicidal ideation which is higher than reports for the general population in Iran (12.7–14%) (Heidari et al. 2024; Karimi et al. 2022) and higher than reported in other countries (Kalb et al. 2019; Mikula et al. 2020). Moreover, the results of a systematic review study in 2020 showed that the prevalence of suicidal ideation in the MS population around the world was calculated at 13% (Kouchaki et al. 2020). This phenomenon of higher SI in patients with MS is multifactorial. In this regard, higher stress levels, lower quality of relationships, and feelings of uncertainty play important roles (Alsharie et al. 2021; Feinstein et al. 2014). Another major underlying cause could be the wide range of stigmas that patients with MS experience during their lives (Cadden et al. 2018). This consequently increases the rate of SI, as a bidirectional association has been observed between SI and stigma (Carpiniello and Pinna 2017). In our population in Iran, cultural and healthcare system complexities and differences with other countries might lead to the observed higher rate of SI in patients with MS. It has also been observed in a meta‐analysis that found an overall prevalence of depression and anxiety to be 47% and 51% in Iran, respectively (Shafiee et al. 2023). In comparison, the worldwide prevalence of depression and anxiety has been estimated to be 23.7% and 21.9%, respectively (Marrie et al. 2015).

While some studies indicate that other sociodemographic characteristics like age, education, smoking, and body mass index could increase SI in Iran (Bazmi et al. 2024; Ekramzadeh et al. 2012; Lalmohamed et al. 2012; Sousa et al. 2021; Ziaei et al. 2017), the results of this study showed no significant correlations between sex, education, smoking, alcohol consumption, disease duration, MS subtype, type of drug used for MS, and walking power with suicidal ideation. The lack of a significant difference in SI between men and women is contrary to the Asgarian et al. study that indicated that women have a higher rate of suicidal thoughts compared to men, which correlates with studies administered on the general population (Asgarian et al. 2020). This might be attributed to differences in study populations and settings. Future studies should be performed to separately compare men and women with MS in terms of SI and their related factors (Saberi‐Zafaghandi et al. 2012). Moreover, there was no significant difference in MS subtype and disease duration in patients with and without SI. This emphasizes the fact that depression and SI could be associated with disabilities rather than disease duration, and hence, in clinical practice, disability control should be prioritized not only to enhance the quality of life but also to prevent SI.

In our study, age had a significant association with SI both in univariable and multivariable models, with younger ages having higher rates of SI, which was similar to Turner et al.’s findings (Turner et al. 2006). Young MS patients often experience short‐term physical disability and psychological disorders, which are associated with an increased risk of suicidal ideation (Brønnum‐Hansen et al. 2005; Feinstein and Pavisian 2017; Janssens et al. 2004; Sariaslani et al. 2021). The study also indicated that there is a strong protective effect of marriage for SI. This result could be attributed to the cooperation of family members, from receiving physical and psychological support since family support in Iranian culture could effectively decrease suicidal tendencies (Ghafari et al. 2015). In this regard, not surprisingly, SI correlated strongly with living alone, and a trend was found for single patients to harbor more thoughts of self‐harm. However, it was the living status of patients with MS, probably a more sensitive index of social isolation and vulnerability than marital status, particularly in a physically disabled population, that emerged as an independent predictor of suicide attempts.

In the univariate model of this current research, employment was a significant protective factor. In this regard, MS patients without any specific job who spend a great amount of time at home are less involved with workplace challenges and consequently experience a higher rate of SI. This observation is in line with a meta‐analysis study that showed employed individuals with MS had better physical and mental health, higher quality of life, and a lower rate of depressive symptoms, compared with unemployed peers (Dorstyn et al. 2019). It should be noted that after adjusting for other variables, having a job showed no significant correlation with SI. Hence, further longitudinal studies are required to obtain more accurate results in this regard.

One of the main observed findings in our study was the significant association between SI and prior suicidal attempts, to the extent that patients with previous suicide attempts had more than double the rate of others. This could be partly explained by the observation in the general population, in whom a previous suicide attempts is the most critical risk factor for suicidal ideation and attempt (Bostwick et al. 2016). Thus, informing about previous suicide attempts or aborted or interrupted attempts is crucial, as a history of such attempts is associated with an increased risk of future attempts.

The present study identified a significant association between SI and ambulatory disability in the univariate model (OR 2.10, 95% CI 1.22 to 3.62, *p* = 0.008) in MS patients, consistent with the Tauil et al. study (Tauil et al. 2018). The low level of suicidal ideation in patients with less movement power might be due to the fact that patients with MS have a higher rate of acceptance of the existing conditions of the disease and try to adapt to them to their situations. However, in the final adjusted model, there was no significant association between SI and walking ability. This finding lowers the importance of measuring of physical disability in the assessment of future SI risk, in comparison with other major predictors such as depression, anxiety, and previous suicide attempt history. In contrast to our findings, the study of Lewis et al. showed that different kinds of disability were associated with suicidality (Lewis et al. 2017). These differences could be attributed to different methodologies utilized in data collection. Noteworthy, the frequency of disease manifestations and presentations, such as duration of disease, subtypes of MS, and type of drug used for MS did not differ significantly between the patients with and without suicidal thoughts which was in contrast to Pompili et al.’s results (Pompili et al. 2012). However, suicidal thoughts may be influenced by the severity and speed of symptom progression and disability in MS patients and require further longitudinal studies with more accurate results in this regard.

Depression is a major determinant of quality of life, and its effects on the lives of patients are widespread and significant. This mood causes an increasing level of disability in MS patients due to decreased functional ability and could, directly and indirectly, affect the prognosis of individuals (Sariaslani et al. 2021). The study by Feinstein et al. indicated that approximately 29% of patients with MS think about suicide, and severe depression could accurately predict 85% of suicide attempts in this population (Feinstein et al. 2014). However, biological mechanism of high rates of depressive symptoms and SI in MS patients is not clear yet. It is supposed that this is due to the biological changes in the brain that activate the psychosocial stress stimulators and disabilities associated with chronic diseases (Sariaslani et al. 2021). Physical illnesses with simultaneous psychiatric diseases may make patients feel that they cannot control their disease, and as they have a lot of difficulties, they are commonly faced with misinterpretations of the nature of their disease (Feinstein 2002; Sariaslani et al. 2021). Also, in line with our findings, a study by Kavaliunas et al. represented a strong association between the severity of depression and anxiety with SI levels in MS people (Kavaliunas et al. 2021).

Our study has several implications for healthcare policymakers and clinicians in Iran. First, the high prevalence of SI in our study in Iran calls for immediate interventions aimed at reducing depression and suicide in patients with MS. Furthermore, clinicians should be aware of psychiatric disorders in addition to neurological problems that these patients may suffer. Hence, it might seem necessary for psychiatric evaluations be added to routine clinical examinations in MS settings. These include, but are not limited to, evidence‐based interventions such as antidepressant administration, cognitive‐behavioral therapy (CBT), support groups, or digital mental health tools. Healthcare providers might also benefit from multidisciplinary collaboration between neurologists, psychiatrists, and psychologists, early psychiatric assessment in younger patients with MS, and a focus on patient education in order to overcome this challenge in this vulnerable population.

To the best of our knowledge, this is the first study exploring the associations between different levels of SI and depression and anxiety levels in Iran. Furthermore, suicidal ideation and depression levels were assessed using two questionnaires (PHQ‐9 and Beck Scale for SI levels) in two steps: screening and whole SI scores. However, some limitations should be noted. First, the cross‐sectional nature of the study did not allow for the evaluation of causal relationships between SI and other variables. Second, the use of PHQ‐9 as the measure of depression assessment might have limitations, especially in reflecting SI, which could be multidimensional in nature. We chose this questionnaire due to it being widely used, which allows us to compare it with previous literature. Also, data were collected from eligible MS outpatients who were attending the clinic, and the findings cannot be generalized to all patients with MS. This also may over‐represent patients receiving care, who may account for lower SI prevalence compared with poorly supported populations. So, future prospective studies with larger sample sizes should be conducted to identify all confounding factors for assessing the association variables with SI in MS patients and its subtypes.

## 5 Conclusion

We found significant associations between lower age, a history of suicide attempts, depression (*p* < 0.001), and anxiety (*p* = 0.05) with SI. Also, considering of direct strong correlation of depression and anxiety scores with SI in both steps (screening part and whole scale), more effective suicide prevention strategies and suicide assessments should be designed as a part of the clinical evaluation in MS patients. On the other hand, MS patients are highly prone to suicidal thoughts and even suicide attempts, and, in this regard, providing effective training and preventive psychological interventions to high‐risk patients, including young patients, patients with history of suicide attempts and patients with psychological disorders, is recommended.

## Author Contributions

**Elham Bazmi**: Conceptualization; writing–original draft; formal analysis; visualization. **Amir Hossein Behnoush**: Conceptualization; writing–original draft; formal analysis; visualization. **Sharareh Eskandarieh**: Writing–original draft; writing–review and editing. **Abdorreza Naser Moghadasi**: Writing–original draft; writing–review and editing. **Samira Navardi**: Writing–original draft; writing–review and editing. **Saeideh Ayoubi**: Writing–original draft; writing–review and editing. **Behnam Behnoush**: Writing–original draft; writing–review and editing. **Mehdi Forouzesh**: Writing–original draft; writing–review and editing. **Mohammad Ali Sahraian**: Writing–original draft; writing–review and editing; conceptualization; data curation; supervision.

## Ethics Statement

The study was approved by the Ethics Committee of Tehran University of Medical Sciences, Tehran, Iran (IR.TUMS.NI.REC.1402.068), and was conducted in accordance with the Declaration of Helsinki. All participants had written informed consent.

## Conflicts of Interest

The authors declare no conflicts of interest.

### Peer Review

The peer review history for this article is available at https://publons.com/publon/10.1002/brb3.70458

## Data Availability

The data used in this study is accessible upon reasonable request from the corresponding author.
